# Supplementary material for: Comparative Study of the Preparation of High-Molecular-Weight Fibroin by Degumming Silk with Several Neutral Proteases
Source: Polymers (Basel). 2023 Aug 12;15(16):3383. doi: 10.3390/polym15163383 (PMC10459046; doi:10.3390/polym15163383)
Supplement: Supplementary file 1 [file polymers-15-03383-s001.zip › polymers-2530477-supplementary.pdf]

Supplementary materials

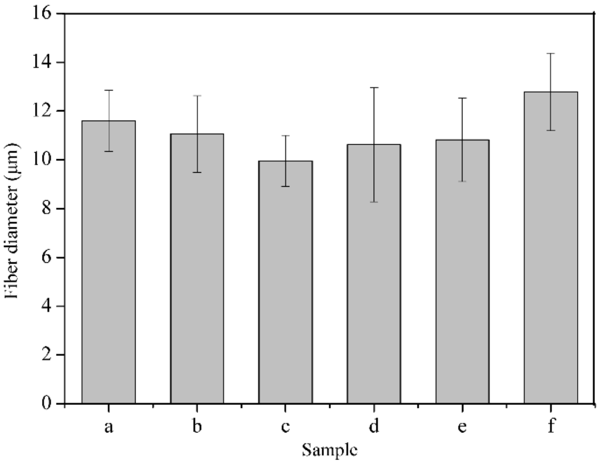

Figure S1. The diameters of degummed silk fibers and raw silk. (**a**) subtilisin-silk; (**b**) trypsin-silk; (**c**) bromelain-silk; (**d**) papain-silk; (**e**) Na_2CO_3-silk; (f) raw silk.
